# The Daily Grind of Living With Chronic Pain: An Applied Hermeneutic Exploration

**DOI:** 10.1177/23333936221148591

**Published:** 2023-01-10

**Authors:** Richard B. Hovey

**Affiliations:** 1 McGill University, Montreal, QC, Canada

**Keywords:** chronic pain, hermeneutics, lived experience, suffering, self-healing, Canada

## Abstract

The purpose of this research is to explore the philosophy regarding understanding the complex experience of living with chronic pain. As well, this article addresses a person’s suffering as an evolving process of learning to not only manage pain but to learn how to live well through exploring their suffering narrative. A hermeneutical interpretive approach was used to engage participants in this research and to offer a philosophical reinterpretation of living with chronic pain from a humanistic and tacit perspective. This work is offered to invite and extend our discussions about the complexity of living with chronic pain. It can also be understood as a process of rewriting oneself from a lived chaotic state of pain into a new affective historical consciousness. This transition from acute to chronic pain explored through a philosophical context can provide insight into the ways in which patients learn to live well with their condition.

## Introduction

The title of this manuscript refers to the “difficult grinding out,” of carrying out routines, and possibly the monotonous tasks required during our daily lives. These vary from the mundane to the extraordinary, which become intensified and more complex when accompanied by chronic pain. We acknowledge with an understanding that living with chronic pain is a multifaceted, multifarious, and often a solitary experience, which requires acknowledgment through multiple narratives of the lived experiences of living with chronic pain (Furnes et al., 2015; Hovey, 2020, 2022). This is explained succinctly through a report from the Canadian Pain Task Force (2020):

An estimated 7.63 million, or one in four Canadians aged 15 or older, live with chronic pain - a condition that although often invisible, is now understood as a disease in its own right. It is often interwoven with other chronic conditions and can affect people across their lifetime. Chronic pain has significant impacts on physical and mental health, family and community life, society, and the economy, with the total direct and indirect cost of $38.3 to $40.4 billion in 2019. (p. 7)

Chronic pain is sometimes defined as pain lasting for more than 3 months (Treede et al., 2015). Persons suffering and transitioning from acute to chronic pain become confronted often not only by a new limitation in their capacity to execute their day-to-day activities, but also report how it affects their relationships and may suffer from social isolation and loss of meaningful employment (Furnes et al., 2015). Additionally, living with chronic pain is understood to increase the likelihood of suicidal ideation, behaviors, and attempts (Racine, 2018). Given the prevalence of chronic pain, healthcare professionals are now more than ever encountering persons and their families experiencing these multi-dimensional challenges. Current literature highlights the challenges of living with chronic pain as measured with validated numeric scales (Consonni et al., 2021; Hadi et al., 2019; Rapti et al., 2019). However, having a numeric representation of pain also means the need to find healing along the journey of living with chronic pain (Toye et al., 2021). Few studies employ philosophical methodologies that encourage research participants to speak freely about their experiences, using their own words and metaphors to reflect and make meaning out of them with the interviewer, an “interpretation of an interpretation” (Davey, 2006, p. 1).

The intent of this research endeavor was to utilize applied philosophical hermeneutics in a philosophical exploration of the experiences of living with chronic pain, encouraging reflexively and personal interpretation, rather than one focused on the biomedical aspects of chronic pain. The type or extent of pain was not the concern of this research but rather it was focused on the meaning of the lived experience of pain and learning to not only manage it but how to learn to thrive. One of the plethora of purposes brought forward through qualitative research is to educate and inform others about a condition they have not experienced first-hand, such as living with chronic pain (Hovey, 2018a, 2022).

The experiences presented in this manuscript may serve to help people living with chronic pain validate their experiences, help nurses to relate to how chronic pain can be expressed by those who live with it beyond their time-limited medical appointments. Additionally, to help those working on research teams with “patient consultants” to better understand their perspectives and how their contributions are irreplaceable (Hovey, 2022; Hovey & Paul, 2007; Hovey et al., 2020).

The author has two decades of experience in performing qualitative research using interpretive approaches such as interpretive phenomenology and applied philosophical hermeneutics. In addition, the author has lived with chronic pain for a decade and has been diagnosed with cancer that affect his overall experience of living with chronic pain. As a qualitative researcher and a person who lives with chronic pain, the author offers reflective insight into the experience of pain through interviews with research participants. This becomes a strength of applied philosophical hermeneutics that results in interviews that are populated both by the participant and the researcher, as each one can relate to the daily challenges of the other and further the exchange and expand understanding.

## Research Approach and Design

This research focused on the experience of the daily challenges of persons living with chronic pain. The study was guided by Gadamer’s (1989) tenets of philosophical hermeneutics following the methodological as described by Moules et al. (2015) and Hovey et al. (2022). The study was approved by the McGill University Research Ethics Board. IRB Study Number A06-B31-13A with the title, “*Experience of living with chronic pain and patients’ experiences of its treatment, future outcomes, and social implications.*” The participants for the study were recruited through posters affixed on the waiting room bulletin board of the McGill University Health Centre, specifically with the Alan Edwards Pain Management Unit. The Alan Edwards Pain Management Unit is an innovative example of a multidisciplinary approach to pain treatment, utilizing a team of 25+ physicians, physiotherapists, psychologists, nurses, and other specialists. Staff work collaboratively in helping people manage their pain through a wide range of approaches, from physiotherapy, psychology, and medication to group therapy as well as complementary and alternative medicine. The posters invited people living with chronic pain to contact the researcher, who would screen them for eligibility, obtain informed consent, and schedule them for an interview. Twenty-five participants, 10 men and 15 women agreed to participate and were individually interviewed. The age range of participants was from their mid-30s to mid-70s at the time of the research project. They represented a wide range of chronic pain conditions and have lived with chronic pain from 2 to 25 years. To be eligible to participate in this study, interested people had to be over 18 years old and have received an official diagnosis of or associated with chronic pain. Pseudonyms were assigned to preserve confidentiality.

The interviews were open-ended/semi-structured, consisting of one key, broad, open-ended question: what does it mean to live with chronic pain? What ensued was an unstructured conversation between the researcher and the participants, including questions and prompts more specific to the experience narrated in the answer of the participant (Hovey et al., 2022). The experience of the interviewer with chronic pain allows them to engage with the participants with almost immediate proximity. The interviews were recorded, transcribed verbatim into text, and analyzed interpretively according to philosophic hermeneutic tradition as outlined by Moules et al. (2015) and Hovey et al. (2022). The text created from the transcribed interviews was read and re-read, with the researcher reflecting on how the parts of the text relate to the whole of all of participants, with the application of the hermeneutic circle (Grondin, 2016). Emerging interpretations were reinforced with relevant chronic pain literature with as appropriate philosophical and non-academic literary works. The culmination of this intensive interpretive work is intended to extend, disrupt, and transform our understandings of the topic.

## Applied Philosophical Hermeneutics

Gadamer, a philosophical hermeneutic scholar, referred to hermeneutics as the “art of understanding.” Understanding is a transformative reflective practice that may open-up new ideas and perspectives and broadens our understanding of a topic (Davey, 2006; Grondin, 2002). There is an understanding that each voice within a topic, including the researcher’s, arrives with a historical situatedness that is contextually and ontologically driven. Philosophical hermeneutics is concerned with how these experiences play out and manifest in our research participants’ lives. In other words, how the participants navigate their new life circumstances (Davey, 2006).

The process of going from a transcribed interview to an interpretation of value and resonance that can extend or overturn current understandings of a topic is challenging to articulate (Hovey et al., 2022). The metaphor of the fusion of horizons can be of assistance in looking between the whole and the parts. It invites a consideration of the particulars of a topic in the context of the familiar, while also attending to the familiar in the context of the particulars (Gadamer, 1989; Grondin, 2016; Moules et al., 2015). This means that certain findings were then further developed, through reading and re-reading the transcripts, to identify other data that expanded the topic. This movement in and out of the data allowed for consideration of findings that might not have been initially visible.

## Interpretive Research Findings

Hermeneutics as a research approach is at its core an interpretation of an interpretation. This means we listen and read the narratives of research participants who help us understand their unique perspectives. These are then interpreted by the researcher into findings that help to further our understanding of complex human experiences. Through a hermeneutical approach, research findings may include literature from outside sources that help in creating an understanding that has depth and is accessible for the reader. The following section endeavors to offer the reader an opportunity to learn from the other, the same topic but from different perspectives.

## On Becoming Other to Oneself: Living in Uncharted Territory

During the research interviews, one of the participants offered a poem he had written exploring his life with chronic pain:

*One day without warning life as I knew it went into mourning.*

*Once the dust had settled, and my new life started, I found myself on a path that was completely uncharted.*

*My days became empty, stagnant, and uneventful, with nothing to look forward to I became very resentful.*

His poem reflects how a life lived with chronic pain begins to fragment one’s previously held identity. Life becomes radically different and uncharted, and within that, one’s relationship with self is altered. A new life is emerging that is radically different and can feel quite distressing. Griffiths, (2016) wrote, “Poetry can heal– it helped me through depression.” She continues, “*I don’t normally write poetry, but in this illness, I could write nothing except poetry. I never normally write at night, but I could write only in darkness.*” This speaks to the need to find ways to express oneself and to find modes of expression to explore suffering to help make sense of it and learn to live with it, even if it disrupts our previously held sense of self.

Ricoeur (1992) wrote that there is a “*dialectical tie between selfhood and otherness*” (p. 3). *Selfhood* is the quality that constitutes one’s individuality, alterity, the state of having an individual uniqueness and *otherness* as being or feeling different in appearance or character from what is familiar, expected, or generally accepted. Pain creates otherness of self from different perspectives, some immediate and others that erode selfhood over time. An example of this phenomenon was provided by one of our research participants:

*My pain is now chronic for almost 6 years. I live within varying degrees of exhaustion. It has profoundly affected my productivity at work. It has created a forced social isolation because I am too tired to meet people. I no longer recognize who I am.*

The transition to a life with chronic pain reveals its disruptive dialectical nature through this research participant’s words. It is first felt physically as a sensation of new pain experienced by the body and then the ensuing suffering created by the multitude of losses, isolation, and fatigue. There are different kinds and intensities of losses that may be experienced by persons living with chronic pain. However, perhaps the most devastating is that of losing one’s sense of self (Hovey, 2018a, 2020). One’s selfhood becomes challenged by an ever-encroaching unfamiliar sense of otherness (Ricoeur, 1992).

*I do not know who I am anymore! I have lost so much to my pain. I cry, I pick myself up again and again, hoping and praying that tomorrow will be better. All I know is that learning to live with this pain will be a lifelong struggle, but the alternative, suicide cannot ever be an option.*

This participant’s emotional expression offers insight into the intensity of loss. However, her narrative also holds hope for a better future even though she knows it will be a lifelong struggle. So intense is the relentlessness of their pain it can result in a person contemplating suicide as a final and permanent solution. Fortunately, the Alan Edwards Pain Management Unit specifically has resources to help with psychological and mental health issues, as described by this participant.

### Drowning in the Wake of Suicide

As a hermeneutic researcher, I do not separate objectively or otherwise by personal life experiences from those of my participants. Some research participant narrative resonates stronger than others as each of us has our own unique, historically effected consciousness, where personal understanding is, essentially, a historically effected series of experiences (Gadamer, 1989). As such mine includes suicide, and perhaps when the participant told me, “*[All] I know is that learning to live with this pain will be a lifelong struggle, but the alternative, suicide cannot ever be an option*,” because of suicide’s far-reaching consequences on other people who are close to you. The author offer’s his reflections on surviving suicide:

*There are multiple fatalities to suicide: the person who dies achieve relief from the pain and suffering that motivated their own death, and the survivors, the bereaved who suffer and grieve must learn how to live, somehow, again. Memories of suicide, where lives may break off, abruptly provoke my own experiences of my mother’s suicide, one which has profoundly affected my personhood, it isolates, blames, shames, and disbelieves. It generates a pain that assaults the very core of one’s existence. Suicide creates a sense of nothingness like no other life event before or ever after has done. All memories of my mother, good or troublesome, begin well but are quickly tainted by my afterlife with her suicide. Remembering my mother is, like putting your hand in a pocket full of razor blades. It’s very painful and people don’t necessarily see the little, small cuts, as I bleed. I have learned that if I want or need to remember her, I need to realize and acknowledge the pain that will be associated with the simple act of remembering someone I loved dearly*.

## We are Our Stories

Frank (2013) writes about the need for telling one’s story and how qualitative research offers this opportunity to research participants.

*The wound is a source of stories, as it opens both in and out: in, in order to hear the story of the other’s suffering, and out, in order to tell its own story. Listening and telling are phases of healing: the healer and the storyteller are one. The healing may not cure the body, but it does remedy the loss of body-self intactness that Cassell identifies with suffering. (p. 183).*

People living with chronic pain must navigate a world now filled with pain, clinics and multiple medical appointments while learning how to live with ambiguity, and yet without becoming paralyzed by this uncertainty. This dialectical reflexivity created by chronic pain requires them to live in uncharted territories, where the whole person may find themselves fragmented and distanced from their previously understood self.

## Fragmented Lives

*The older I get the worse it [chronic pain] started to get, and I would have anxiety with that, brain fog, not be able to think ‘um’ and just have little concentration, it just got worse and worse and worse and worse. Just pain everywhere kind of like a tight elastic like if you had a body suit on that was really really tight and all you wanted to do was unzip it and get it off. That’s what it would feel, feel like, that’s what it feels like.*

As this participant expressed through a metaphor of pain feeling encased in a tight elastic suit, it suffocates, incarcerates, and is omnipresent. We begin to get a sense that this pain is very different from other expressions of pain we may have previously experienced. The image of being trapped in pain and the impossible wish to find relief by unzipping a suit and stepping out of it qualifies this pain as all-encompassing. This participant reveals their experience with pain to be unimaginable until it happened to them, and they had to invent a metaphor to discuss it with others.

*So, everything - my whole life changed to just being able to walk and ride a bicycle for a little bit. That’s about it. So, all my other sports and activities are gone out the window.*

Windows as metaphors can represent different interpretations, letting light in, being opened for fresh air, or protection from the elements. From a human perspective, the eyes are sometimes referred to as the windows to our souls. The window from this perspective of self-looking outwards expresses the loss of one aspect of their pre-pain life as, “out the window.” They feel now more like a spectator of their previous life rather than an active participant in their new life living with pain.

*“I have been told that people can see the pain through my eyes”*

Finding the words that reflect these intense feelings becomes one of the challenges associated with living with chronic pain. We found that multiple participants used metaphors to express their experiences. However, the challenge often becomes how and where someone in chronic pain starts to create their personal pain narrative. Perhaps by picking up the fragments one by one they can begin to understand what this new life might feel like, chaotic at first, but ever evolving. For some of the research participants turning to literature and poetry helped to begin this process. This passage from Rothwell’s (2013) Belomor touches on this sense of immediate loss and chaos in confronting chronic pain:

*The way you saw the world lying in fragments – everything lost, and wrecked, and scattered; and the task of life was in collecting up those fragments, looking, seeking for the resonances and the echoes – the shards – you said what was left to us has been exploded, pulverised, reduced to rubble; no more order, nothing, no more structure or harmony, no sequence – and the hardest thing’s to find words that fit together, that hold any truth at all. (p. 215)*

Through telling their stories, research participants began to examine their lives living with chronic pain as the way they view their personal fragments scattered around them, relationships, work, education, family, and hope. Their prior lives are thrown out the window, lost and shattered. Narratively, they have begun to create a new identity, one of suffering at the beginning that enables them to place the pieces of a new life back together. The idea of a life fragmented makes sense as they explore loss from a new and completely different perspective. Through self-reflection and interpretation, their fragments may be re-storied to offer the possibility of a new perspective on life.

Hermeneutic interviews are open-ended and offer the participants an opportunity to tell their stories, uninterrupted and without time limitations to explore their experiences. Some of the interviews ebbed and flowed back to childhood memories of adapting to challenging situations, relationships, work, and future prospects, interweaving pain within their stories of pain (Hovey, 2022).

For one participant, a thread carrying their narrative was a new sense of self re-negotiated with adaptation and acceptance of his life. For him, this adaptation was found in locating himself among others who shared chronic pain. This participant found a place of strength by saying,

“*[b]ut I can still walk so I’m still smiling you know? I mean people are worse off than I am.*

This participant chooses to not end his narrative by being portrayed as a victim but rather, to reveal his inner strength and character when he says that he can still “smile” and acknowledges that there are people who are “worse off.” In this way, the narrative ends with how a person suffers but still wants to live his life, even with pain, with concern for others worse off.

## The Wisdom Through Suffering

*For many years’ distraction was the best way out of pain for me. I did distraction like a drug. . . But tuning pain out was only a temporary solution for me, and eventually I had to face my pain in order to find a real solution.*

*I was 43 when I had the injury that caused the chronic pain, and now I just turned 71 and I have the least amount of pain I’ve ever had. I haven’t stopped living life intensely, but I’ve certainly toned it down. Although I don’t want to do it, I am now tuning the pain back-in, so I can listen to it.*

This participant provides an example of how we may become wise through suffering, *pathei mathos* (Davey, 2006; Hovey, 2018b). Her life with pain demonstrated how someone who was working through her own process of deception and undeception (Gadamer, 1989) over many years, the acknowledgment of her pain and how it affected her life could no longer be tolerated as she surrendered to the reality of her pain. This thinking also aligns with Gadamer’s idea of historically affected consciousness (Gadamer, 1989) as it expresses our embodiment of our story with a particular history and enculturation that contributes to our socially constructed identity. Our identities are part of a complex process of all of our experiences, culture, education, social interactions, employment, etc., and their interpretations into our unique life contexts, status, and sense of self.

Another participant’s story was one of suffering, grief, and loss. Gaining wisdom through experience is a personal journey that is unique and will be live through with time (Hovey & Amir, 2013).

*You have to take on so much almost-intolerable loss and suffer so much almost-intolerable grief, to wipe out who and what you were before you can get through the process, and you are very lucky if you can survive it.*

People suffer because of the human capacity for self-reflection, awareness, and the ability to sense loss. Whereas grieving is a human response to profound loss, suffering can be mitigated. We suffer from our search for meaning and to make sense of our situation (Kearney, 2003). The anguish that a person experiences amidst the continual slow deteriorating loss of one’s world is profound (Hovey, 2016). Pain and suffering disrupt and fragment our projected meaning of a continual process of self; suffering roadblocks our existence into another form of becoming renewed. As another participant expressed,

*I think it’s important because every person whether they think they’re an artist or not because if you’re a person you’re creating even if you don’t have something, a physical thing or you know to show for it and then you can share that with other people. So, I feel like my journey which hasn’t ended but it it’s ‘uh’ in being able to be there for myself and to have the acknowledgement of this pain and everything I’ve been able to help other people through music.*

Suffering, from its etymological origins, also means “undergoing of punishment” because pain can feel like a punishment although we did nothing wrong to deserve this condition. It differs from pain in that, as Cassell (1999) says, it is a specifically human phenomenon; bodies can experience pain but only people suffer, and everyone suffers differently.

This adds an additional layer of complexity to understanding the experience of chronic pain, related suffering, and how individuals interpret their pain based on how it affects their lives not strictly as a factor of the pain intensity per se but rather understood through the meaning, intensity and perception of the losses they have experienced within their lives.

Suffering is an affliction of the totality of the person in pain, not merely their physical bodies, but pervasive through multiple dimensions of health and well-being such as physical, emotional, relational, recreational, occupational, environmental, intellectual, psychological, social, or spiritual expressions of suffering (Hovey, 2012). Medical interventions are not specifically designed to confront suffering but rather to remove or numb the pain. In the case of chronic pain, the treatments themselves may become the source of suffering when pain persists, and treatments fail.

Suffering of this sort can be called “iatrogenic” in that it is suffering caused by nurses themselves, when unable to distinguish properly between a person’s pain and their suffering. While pain can be the cause of suffering, it can only act as such to the degree that we allow it to do so, for there is a crucial difference between pain and discomfort and the distress we might experience over feeling pain or suffering (Cassell, 1999).

The chronically pained person experiences the world as a unique and difficult venture, which over time transforms their sense of themselves as their personal narrative may become repetitively situated within the pathology of their pain (Hovey, 2016). The suffering person should also be able to talk about their suffering, rather than exclusively about treatments, pharmacology, and the appointment with the next specialist. This kind of narrative is ontologically oriented and becomes means to cathartically work and talk through their suffering to begin to understand it within a unique re-contextualization of their life with pain (Madison, 2015). The personal narrative can be an effective response to suffering, it is to engage with the suffering person about their suffering through reflection and explication, rather than a reductionist factual narrative about their pain (Kearney, 2003).

## Re-Storying Opens-Up to New Possibilities

Rothwell (2013) offered that using our sensibilities, our ability to sense, in order “*to find the scattered pieces that belong together*” (p. 31) through the stories told of our lives, however chaotic at first, will help heal through narrative medicine and stories.

Sometimes our stories must get worse before getting better as a progression of making sense of suffering, through the transitioning or sorting out of these fragmented stories, which takes time, even at first somewhat incoherently. This is another opportunity to explore why we need to understand both pathological and non-pathological narrative accounts within the same life. Pain needs to be contextualized because of its pervasiveness into all aspects of a person’s life which means that chronic pain is far more complex than a medical problem to resolve. Otherwise, we risk limiting the suffering person to the brevity of their narrative that is primarily guided by their experiences from healthcare. Qualitative research offers this opportunity to be undone so that another narrative may emerge, one more capable of addressing the true tasks of healing their suffering. Reading qualitative research will provide insight for nurses and researchers about conditions they only understand from a medical or theoretical perspective and allow them to include other ways of knowing and understanding. The lived experience of the person living with chronic pain is reflective, relational, and interpretative. Learning from the other may be challenging for people not used to reading outside of their discipline-specific literature. However, we are reminded that chronic pain is a topic of interest for everyone and that as we expand our horizons of understanding, we may become more holistic researchers and nurses (Hovey, 2022).

Chronic pain needs a different approach to understanding pain as a whole life-altering experience. The suffering person may need to take their chaotic thinking about life and pain and relearn to construct a new live narrative through self-empathy and compassion (Hovey, 2016). Philosophy understands empathy as a moral, relational emotion; the very form of attachment seen to be necessary for living responsibly together. This emphasis on what empathy brings to our sense of togetherness is why the promise of empathy comes to the forefront for our communication with pain sufferers (Todd, 2003). Rossiter enters this conversation about living with chronic pain through her work in learning in adulthood through the concept and process of “possible selves,” which advocates that we construct and can re-construct a narrative understanding of identity. However, this can also be renegotiated if someone is aware of this process and is willing to be transformed. In other words, the suffering person adopts a new sense of self that is understood as another unfolding story of possible selves, one not confined by their pained existence. We can understand the development and elaboration of “possible selves” as a process of self-storying (Rossiter, 2007). The relevance of this concept from the adult learning literature is found in its potential to help the suffering person to re-story their life and learn how to articulate new narratives that enable or assist the person to see, sense, and create a new vision or a transformation in their life. Randall (1996) has described transformative learning as the process of “restorying” oneself into a new identity. A key component of the narrative understanding of identity development is the utility of self-authoring. This concept gives hope to the suffering person because it implies that individuals can exercise some choice in guiding their own development and shaping their own life narrative, through story.

Narrative self-learning has the capacity, according to Kegan (2000), to change the epistemological perception of self from being one acted on or being trapped by external influences of our suffering, to acting on or narrating anew as an ontological repositioning of the self with their suffering. The main point here is that personal narratives have the capacity for empowering in their attention to self-authorship. To the extent that we understand the identification, elaboration, and motivation to realize possible selves as part of the self-storying process, we can appreciate the role of possible selves in the construction as well as the reconstruction of the life narrative.

## Concluding Thoughts

The intention for writing this manuscript was to demonstrate that pain and in particular chronic pain should be understood from multiple perspectives not only for the person living with chronic pain but to offer insight for nurses and researchers. The participants in this study discussed from their own perspective the amount of effort needed to live well with their condition, resulting in their “daily grind” being the work of managing their pain. With growing attention to patient experience and perspective, engagement research within both the clinical setting (healthcare) and the community, where lives are lived requires a reconciliation of the scientific explanatory (epistemology of pain) and the philosophical reflective (ontology of pain) together, such that healthcare is always reminded of the person living with a variety of chronic pain (Hovey et al., 2016, 2020). Developments in undergraduate and pre-licensure curricula have highlighted the usefulness of narrative medicine to prepare future nurses for more meaningful clinical encounters (Milota et al., 2019). Still, the need to encourage current practitioners to read qualitative research persists (Hovey, 2022).

The author of this manuscript endeavored to shift our understanding of pain from a predominantly medicalized topic to one that is about the people who live with chronic pain. Together, through this applied hermeneutic research with people living with chronic pain, we explore thinking with the pained person about their experiences. It involves the suffering person’s intuitive sense of what remains underdeveloped in our understanding of life and chronic pain, not as an impossible journey of learning to live well with chronic pain, but one that slowly evolves over time.

This manuscript interweaves the personal experiences of pain sufferers, with a philosophical perspective on suffering as a re-storying of pain as suffering, and a hermeneutical interpretation of this intersection of pain, suffering, and understanding through the dialectic of self as other created by pain. Although the daily grind through life continues, we can find inspiration from others who shared their stories, authentically, and sincerely.
